# Microneedle array delivered recombinant coronavirus vaccines: Immunogenicity and rapid translational development

**DOI:** 10.1016/j.ebiom.2020.102743

**Published:** 2020-04-02

**Authors:** Eun Kim, Geza Erdos, Shaohua Huang, Thomas W. Kenniston, Stephen C. Balmert, Cara Donahue Carey, V. Stalin Raj, Michael W. Epperly, William B. Klimstra, Bart L. Haagmans, Emrullah Korkmaz, Louis D. Falo, Andrea Gambotto

**Affiliations:** a Department of Surgery, University of Pittsburgh School of Medicine, Pittsburgh, W1148 Biomedical Science Tower, 200 Lothrop St., Pennsylvania, PA 15213, USA; b Department of Dermatology, University of Pittsburgh School of Medicine, W1150 Biomedical Science Tower, 200 Lothrop St., Pittsburgh, PA 15213, USA; c Department of Radiation Oncology, University of Pittsburgh, Pittsburgh, PA 15213, USA; d Center for Vaccine Research, Department of Immunology, University of Pittsburgh, Pittsburgh, PA 15213, USA; e Department of Viroscience, Erasmus Medical Center Rotterdam, Rotterdam, the Netherlands; f Department of Bioengineering, Swanson School of Engineering, University of Pittsburgh, Pittsburgh, PA 15231, USA; g Clinical and Translational Science Institute, University of Pittsburgh, Pittsburgh, PA 15213, USA; h The McGowan Institute for Regenerative Medicine, University of Pittsburgh, Pittsburgh, PA 15219, USA

**Keywords:** Subunit vaccines, Trimerization, Microneedle array, MERS-CoV S1, SARS-CoV-2, COVID-19

## Abstract

**Background:**

Coronaviruses pose a serious threat to global health as evidenced by Severe Acute Respiratory Syndrome (SARS), Middle East Respiratory Syndrome (MERS), and COVID-19. SARS Coronavirus (SARS-CoV), MERS Coronavirus (MERS-CoV), and the novel coronavirus, previously dubbed 2019-nCoV, and now officially named SARS-CoV-2, are the causative agents of the SARS, MERS, and COVID-19 disease outbreaks, respectively. Safe vaccines that rapidly induce potent and long-lasting virus-specific immune responses against these infectious agents are urgently needed. The coronavirus spike (S) protein, a characteristic structural component of the viral envelope, is considered a key target for vaccines for the prevention of coronavirus infection.

**Methods:**

We first generated codon optimized MERS-S1 subunit vaccines fused with a foldon trimerization domain to mimic the native viral structure. In variant constructs, we engineered immune stimulants (RS09 or flagellin, as TLR4 or TLR5 agonists, respectively) into this trimeric design. We comprehensively tested the pre-clinical immunogenicity of MERS-CoV vaccines in mice when delivered subcutaneously by traditional needle injection, or intracutaneously by dissolving microneedle arrays (MNAs) by evaluating virus specific IgG antibodies in the serum of vaccinated mice by ELISA and using virus neutralization assays. Driven by the urgent need for COVID-19 vaccines, we utilized this strategy to rapidly develop MNA SARS-CoV-2 subunit vaccines and tested their pre-clinical immunogenicity in *vivo* by exploiting our substantial experience with MNA MERS-CoV vaccines.

**Findings:**

Here we describe the development of MNA delivered MERS-CoV vaccines and their pre-clinical immunogenicity. Specifically, MNA delivered MERS-S1 subunit vaccines elicited strong and long-lasting antigen-specific antibody responses. Building on our ongoing efforts to develop MERS-CoV vaccines, promising immunogenicity of MNA-delivered MERS-CoV vaccines, and our experience with MNA fabrication and delivery, including clinical trials, we rapidly designed and produced clinically-translatable MNA SARS-CoV-2 subunit vaccines within 4 weeks of the identification of the SARS-CoV-2 S1 sequence. Most importantly, these MNA delivered SARS-CoV-2 S1 subunit vaccines elicited potent antigen-specific antibody responses that were evident beginning 2 weeks after immunization.

**Interpretation:**

MNA delivery of coronaviruses-S1 subunit vaccines is a promising immunization strategy against coronavirus infection. Progressive scientific and technological efforts enable quicker responses to emerging pandemics. Our ongoing efforts to develop MNA-MERS-S1 subunit vaccines enabled us to rapidly design and produce MNA SARS-CoV-2 subunit vaccines capable of inducing potent virus-specific antibody responses. Collectively, our results support the clinical development of MNA delivered recombinant protein subunit vaccines against SARS, MERS, COVID-19, and other emerging infectious diseases.

**Research in context**

**Evidence before this study**

Coronavirus is an emerging pathogen with exponentially increasing significance due to the high case fatality rate, the large distribution of reservoir, and the lack of medical countermeasures. The public health emergencies caused by Coronaviruses, including SARS-CoV, MERS-CoV, and now SARS-CoV-2, clearly demonstrate the urgency to evaluate candidate vaccines to combat these outbreaks. Continuous research efforts from previous epidemics contribute to the efforts of investigators to quickly develop safe vaccines against these emerging infections; however, the recent COVID-19 pandemic highlights an important continuing need for the rapid design, production, testing, and clinical translation of candidate vaccines.

**Added value of this study**

These studies demonstrate the rapid development and immunogenicity of novel microneedle array (MNA) delivered recombinant coronavirus (SARS-CoV-2) vaccines. MNA delivered MERS-S1 subunit vaccines induced potent and long-lasting antigen antigen-specific immune responses. Notably, MNA delivery of these vaccines generated significantly stronger immune responses than those administered by traditional subcutaneous needle injection, indicating the improved immunogenicity by skin-targeted delivery. These efforts with MNA MERS-S1 subunit vaccines enabled the rapid design and production of MNA SARS-CoV-2 vaccines, capable of eliciting potent virus-specific antibody responses that were evident as early as 2 weeks after immunization. Rapid design and production of MNA-embedded SARS-CoV-2-S1 subunit vaccines using clinically applicable MNA fabrication methods supports the development of MNA delivered recombinant coronavirus vaccines for clinical vaccine applications. Collectively, our studies support the clinical development of MNA protein subunit vaccines for COVID-19 and other emerging infectious diseases.

**Implications of the available evidence**

MNA delivery of coronaviruses-S1 subunit vaccines is a promising immunization strategy against MERS-CoV and SARS-CoV-2 infection and can be adapted for other subunit vaccines against a broad range of infectious diseases. The recent advances in recombinant DNA technology and vaccine delivery strategies enable quick design, production, and testing of vaccines against emerging infections. We plan to evaluate these SARS-CoV-2 vaccines for other important predictors of vaccine efficacy in humans, including the induction of neutralizing antibodies and for their ability to prevent infection in animal challenge models, when these assays and preclinical models become available. Thus far, our studies suggest that it may now be possible to produce clinical grade vaccines against novel pathogens for human testing and subsequent global distribution in time to significantly impact the spread of disease.

Alt-text: Unlabelled box

## 1 Introduction

On December 31, 2019, Chinese authorities in Wuhan announced a cluster of pneumonia cases of unknown etiology, most of which included patients who reported exposure to Wuhan's Huanan Seafood wholesale market. Emergence of another pathogenic zoonotic HCoV was suspected, and on January 9, 2020, the Chinese Center for Diseases Control reported that a novel coronavirus was the causative agent. Shortly thereafter the genomic sequences of several isolates became publicly available. On February 10, 2020, the WHO named the disease, caused by the new coronavirus, COVID-19, and the new virus was named severe acute respiratory syndrome coronavirus 2 (SARS-CoV-2). Genomic analyses indicated that SARS-CoV-2 shares genomic similarities with SARS-CoV within the receptor-binding motif that directly contacts the human receptor ACE2. This has important implications for vaccine design and for predicting pandemic potential. Human-to-human transmission of SARS-CoV-2 has now been established, and the situation with SARS-CoV-2 is evolving rapidly with the numbers of verified cases growing into the thousands. Accordingly, The World Health Organization (WHO) has declared COVID-19 a global pandemic.

Another recently emerged coronavirus, the Middle East Respiratory Syndrome Coronavirus (MERS-CoV) was first isolated in 2012 in Saudi Arabia [1]. As of January 31st, 2020 the World Health Organization (WHO) has been notified of 2,513 laboratory-confirmed cases of infection with MERS-CoV that include 864 related deaths with a fatality rate of 34.4% [2]. Cases have arisen in 27 countries globally, including a severe outbreak in the Republic of Korea in 2015 [3,4]. MERS-CoV is still endemic in the Middle East, most particularly in Saudi Arabia, where more than 80% of cases have occurred. No efficacious drugs or vaccines are currently approved for human use [2,5]. The WHO included MERS-CoV on the list of priority blueprint diseases for which there is an urgent need for accelerated research and development of a safe and efficacious vaccine [5,6].

For both these coronaviruses, the spike (S) protein is crucial for viral transmission and infection, and determines the tropism of the virus and host cell entry. SARS-CoV-2 binds the ACE2 receptor as MERS-S binds to the cellular receptor dipeptidyl peptidase 4 (DPP4) via the receptor-binding domain (RBD) in the N-terminal surface subunit (S1), and then employs its C-terminal transmembrane subunit (S2) to fuse with the host cell membrane. [7], [8], [9], [10], [11] Due to this vital functional property and established antigenicity, the S protein is an important target for vaccine development. Our previous studies have shown that adenoviral vaccine candidates expressing SARS-CoV-S1 and MERS-S1 subunits induced more efficacious antibody-mediated neutralizing activity than full-length S1, suggesting the subunit immunogen as an ideal vaccine candidate [12]. The native S proteins exist as a trimeric form on the surface of the virus. However, when its ectodomain or S1 is expressed as a recombinant protein in eukaryotic systems, the protein exists predominantly in a monomeric form [13,14]. Here, to make trimeric recombinant codon optimized subunit proteins mimicking the native viral structures, we have fused the MERS-CoV-S1 and SARS-CoV-2-S1 segments to a 27-amino acid sequence called the foldon domain, which is a natural trimerization domain derived from the C terminus of bacteriophage T4 fibritin [15,16]. Inclusion of a foldon trimerization domain promotes a trimeric structure and has been shown to increase the immunogenicity of several viral antigens including Influenza virus hemagglutinin, HIV-1 glycoprotein, and SARS-CoV S1/S-ectodomain, supporting the importance of mimicking native viral trimeric structures [14,17,18]. Further, recent work has shown that a recombinant trimeric receptor-binding domain (RBD) of MERS-CoV, made by fusing RBD with foldon domain (RBD-Fd), elicits potent RBD-specific neutralizing antibodies and protects hDPP4 transgenic mice from viral infection [19]. To potentially improve the immunogenicity of these vaccines, we also constructed S1 subunits with integrated Toll-like receptor (TLR) agonist sequences, specifically RS09 and flagellin C. RS09 is a synthetic form of an LPS mimic 7-mer peptide, which binds to TLR4 and induces nuclear localization of the transcription factor NF-κB, resulting in transcription of inflammatory cytokines and secretion of chemokines [20]. Flagellin is a highly conserved natural bacterial ligand that binds to TLR5 to activate TLR5 expressing dendritic cells, neutrophils, lung epithelial cells and pneumonocytes, augmenting immunogenicity in several vaccine models [[21], [22], [23]].

The skin is an ideal target for immunization. It contains a rich population of antigen presenting and immune accessory cells capable of inducing a proinflammatory microenvironment favoring the induction of potent and durable adaptive immunity [24,25]. Of several emerging skin-targeted drug delivery methods, dissolving MNAs have emerged as a minimally-invasive intracutaneous approach [26–28]. MNAs used here are developed from mechanically strong water-soluble polymers to physically breach the outermost layer of skin (stratum corneum) and then rapidly dissolve in the underlying viable epidermis and dermis to deliver cargos to skin microenvironments [27,29].

Importantly, MNA delivery results in high concentrations of vaccine components in the local skin microenvironment, thereby contributing to a dose-sparing effect that could decrease the required vaccine doses for efficacious immunization and substantially reduce both cost and toxicity [27,[30], [31], [32]]. Although the stability of each vaccine candidate in MNAs needs to be validated at different temperatures for varying storage periods, the available literature indicates that MNA-embedded vaccines have the potential to remain stable for an extended period of time without expensive “cold chain” requirements [33,34]. Further, previous animal and clinical studies suggest that MNAs could provide a safe and well-tolerated delivery platform for efficacious immunization strategies [[35], [36], [37], [38], [39]].

Here, we demonstrate the rapid development of MNA SARS-CoV-2 S1 subunit vaccines and their promising immunogenicity in mice developed using our evolving experience with MERS-CoV vaccines and MNAs. First, we evaluate the immunogenicity of trimeric form of MERS-S1 subunit vaccines delivered subcutaneously by traditional needle injection, or intracutaneously by MNAs. Driven by the promising immunogenicity of MNA-MERS-S1 vaccines and the urgent need to respond to the recent coronavirus pandemic (COVID-19), we rapidly (within 4 weeks of the identification of the SARS-CoV-2 S1 sequence) designed and produced MNA SARS-CoV-2 S1 vaccines and tested their immunogenicity in mice. We describe the rapid development of MNA embedded SARS-CoV-2-S1 subunit vaccines using clinically-applicable MNA production methods by relying on our experience with clinical trials utilizing MNA delivery (ClinicalTrials.gov Identifier: NCT02192021). Taken together, our results support the development of MNA delivered recombinant coronavirus vaccines for clinical applications.

## 2 Materials and methods

### 2.1 Construction of adenoviral vectors and plasmids

The codon-optimized gene encoding MERS-S1 (amino acids 1 to 725 of full-length MERS S, according to the GeneBank JX869059) lacking stop codon flanked with SalI & BamHI was amplified by PCR from pAd/MERS-S1 ^12^ and was used to replace the ZIKV-E in in pAd/ZIKV-Efl ^40^ at SalI & BamHI sites fused with BamH I-linked T4 fibritin foldon trimerization domain (f), Tobacco Etch Virus Protease (Tp), and six histidine tag (6H), generating plasmid pAd/MERS-S1f. For the construction of pAd/MERS-S1fRS09 or pAd/MERS-S1ffliC, BamH I-fTp6H-Not I of pAd/MERS-S1f was replaced with codon optimized BamH I-f-RS09(APPHALS)-Tp6H-Not I or BamH I-f-fliC (GenBank ACY88831)-Tp6H-Not I. Subsequently, replication-defective human adenovirus serotype 5, designated as Ad5.MERS-S1f, Ad5.MERS-S1fRS09, and Ad5.MERS-S1ffliC, were made by loxP homologous recombination and purified and stored as described previously [[41], [42], [43]].

For the construction of pmax/SARS-CoV-2-S1, the codon-optimized gene encoding SARS-CoV-2-S1 amino acids 1 to 661 of full-length from BetaCoV/Wuhan/IPBCAMS-WH-05/2020 (GISAID accession id. EPI_ISL_403928) lacking stop codon flanked with HindIII-SalI & BamHI-6H-NotI was synthesized (GenScript) and generated by subcloning the codon-optimized SARS-CoV-2-S1 gene into pmaxCloning (Lonza), at HindIII/NotI sites. For the construction of pmax/SARS-CoV-2-S1fRS09, BamH I-6H-Not I of pmax/SARS-CoV-2-S1 was replaced with codon optimized BamH I-f-RS09(APPHALS)-Tp6H-Not I.

### 2.2 Purification of recombinant proteins

The recombinant proteins named rMERS-S1f, rMERS-S1fRS09, and rMERS-S1ffliC were expressed in Human Embryonic Kidney (HEK) 293 cells infected with Ad5.MERS-S1f, Ad5.MERS-S1fRS09, and Ad5.MERS-S1ffliC, respectively, and purified by His60 Ni Superflow Resin (Clontech) under native conditions from the supernatant as described previously [40]. Briefly, the purified recombinant proteins were treated with AcTEV protease (Life Technology) followed by affinity chromatography on a His60 Ni Superflow Resin to remove six-histidine tags and poly-histidine tagged protease from the cleavage reaction. The cleaved native recombinant proteins were collected from the flow-through fraction. The eluted solution was concentrated and desalted with phosphate buffered saline (PBS) in an Amicon Ultra centrifugal filter devices (Millipore).

For expression of recombinant proteins, rSARS-CoV-2-S1 and rSARS-CoV-2-S1fRS09, 293HEK cells were transfected by electroporation (Celetrix). Briefly, 293HEK cells were counted and suspended with plasmids in the electroporation buffer at 5 × 10^7^ cells, 200μg/ml. The mixture containing cells and plasmids was transferred into 1ml Celetrix electroporation tube with 25 mm distance between the electrodes, and immediately subjected to electroporation under 930V and 30ms, and then incubated for 72 hrs at 37°C in a humidified atmosphere with 5% CO_2_. The recombinant proteins, rSARS-CoV-2-S1 and rSARS-CoV-2-S1fRS09, were purified by His60 Ni Superflow Resin (Clontech) under native conditions from the supernatant as described previously [40]. The concentrations of the purified recombinant proteins were determined by the Bradford assay using bovine serum albumin (BSA) as a protein standard.

### 2.3 SDS-PAGE and Western blot

To evaluate the recombinant adenoviruses, A549 cells were transduced with ten multiplicity of infection (MOI) of Ad5.MERS-S1f, Ad5.MERS-S1fRS09, and Ad5.MERS-S1ffliC. At six hours after infection, cells were washed three times with PBS, and added with serum-free media, and incubated for 48 h. The supernatant of A549 infected with adenoviruses were subjected to sodium dodecyl sulfate polyacrylamide gel electrophoresis (SDS-PAGE) and Western blot. Briefly, after the supernatants were boiled in Laemmli sample buffer containing 2% SDS, or native sample buffer, with or without beta-mercaptoethanol (β-ME), the proteins were separated by Tris-Glycine SDS-PAGE gels and then transferred to polyvinylidene fluoride (PVDF) membrane. After blocking for 1h at room temperature with 5% non-fat milk in PBST, anti-6xHis monoclonal antibody (1:50000) (Invitrogen) were added and incubated overnight with at 4 °C as primary antibody, and horseradish peroxidase (HRP)-conjugated goat anti-mouse IgG (1:10000) (SantaCruz) was added and incubated at RT for 2 h as secondary antibody. After washing three times with PBS, the signals were visualized using ECL Western blot substrate reagents and Amersham Hyperfilm (GE Healthcare).

### 2.4 Fabrication of dissolvable microneedle arrays

Dissolvable microneedle arrays (MNAs) incorporating the protein MERS-S1f, MERS-S1fRS09, MERS-S1ffliC, SARS-CoV-2-S1, or SARS-CoV-2-S1fRS09) were fabricated from carboxymethyl cellulose (CMC, 90 kDa MW) at room temperature (22°C) using our previously described three-stage MNA manufacturing strategy [29,32]. Briefly, the production molds were prepared by casting Polydimethylsiloxane (PDMS, SYLGARD® 184) elastomer onto the micromachined MNA master molds that include 100 obelisk-shaped microneedles in a 10 × 10 array. The height, width, and apex angle of microneedles were chosen to be 750 µm, 225 µm, and 30°, respectively. Next, CMC-based MNA-rMERS-S1f, MNA-rMERS-S1fRS09, MNA-rMERS-S1ffliC, MNA-rSARS-CoV-2-S1, or MNA-rSARS-CoV-2-S1fRS09 vaccines were produced through a two-step spin-drying technique. First, 20 μl of purified protein was dispensed onto each MNA production mold and centrifuged for 1 min at 2500 g to fill the microneedle cavities. Subsequently, the excess solution was removed, and the residual protein solution was spin-dried into the tip of the microneedle cavities of the mold by centrifugation at 2500 g for 30 min. The tip-loaded protein in the production molds was overlayered with 75 mg of CMC hydrogel (20% w/w) to form the mechanically strong microneedles and the backing layer of the MNAs. The molds loaded with the CMC-hydrogel were then centrifuged for 3 h at 2500 g to obtain final dissolvable MNA vaccines. The theoretical loading of each MNA-rMERS-S1f, MNA-rMERS-S1fRS09, MNA-rMERS-S1ffliC, SARS-CoV-2-S1, or SARS-CoV-2-S1fRS09 was 25 µg, 25 µg, 40 µg, 20 µg, or 20 µg proteins, respectively. The geometric integrity of the fabricated MNAs was confirmed through optical stereomicroscopy imaging before the experiments. Importantly, this MNA design and fabrication strategy has also been used for MNAs utilized in our ongoing Phase 1 clinical trials (ClinicalTrials.gov Identifier: NCT02192021). In line with our efforts toward clinical production of MNA SARS-CoV-2 subunit vaccines, we gamma irradiation (25 kGy) sterilized a small group of these MNA SARS-CoV-2 subunit vaccines to determine clinically relevant sterilizing conditions and any effect on immunogenicity.

### 2.5 Animals and Immunization

BABL/c mice (five animals per group) were inoculated subcutaneously with 25 µg of MERS-S1f only, 25 µg of MERS-S1f plus 20 µg of MPLA (monophosphoryl lipid A; TLR-4 agonist), 25 µg of MERS-S1fRS09, and 40 µg of MERS-S1ffliC or PBS as a negative control and 10^11^vp of Ad5.MERS-S1 as a positive control. It is noted that MPLA was added in a single control group at similar doses as used previously to establish a positive control for the adjuvanted subunit vaccines [44]. The MNA rMERS-S1f, rMERS-S1fRS09, or rMERS-S1ffliC vaccines were applied to the abdomen of anesthetized mice and removed after 10 min. Two weeks after the primary immunization, mice were boosted intranasally (i.n.) or intracutaneously (MNAs) with the same dose of the corresponding immunogens.

For SARS-CoV-2 study, C57BL/6 female mice (five animals per group) were inoculated intracutaneously with MNAs loaded with of SARS-CoV-2-S1 or SARS-CoV-2-S1fRS09 protein, or PBS as a negative control. At two weeks after the primary immunization, mice were similarly boosted with the same dose of the corresponding immunogens. To preliminarily evaluate the effects of sterilizing gamma irradiation on MNA-SARS-CoV-2 vaccines, 2 mice per group were irradiated using clinical sterilizing conditions. Mice were bled from the saphenous vein at week 0, 1, 2, 3, and 4, and serum samples were evaluated for SARS-CoV-2-S1 antibody by enzyme-linked immunosorbent assay (ELISA).

Mice were maintained under specific pathogen-free conditions at the University of Pittsburgh, and all experiments with mice were conducted in accordance with animal use guidelines and protocols approved by the University of Pittsburgh's Institutional Animal Care and Use (IACUC) Committee.

### 2.6 FACS analysis

Two weeks after the prime immunization, pooled sera were obtained from all mice and screened for MERS-S-specific antibodies using fluorescence-activated cell sorter (FACS) analysis of Human Embryonic Kidney (HEK) 293 cells transfected with either pAd/MERS-S or pAd control using Lipofectamine 2000 (Invitrogen) as described previously [12]. Briefly, after 36 h at 37°C, the transfected cells were harvested, trypsinized by incubation with 0.25% Trypsin, 2.21mM EDTA, for 2-3 min at 37°C, followed by trypsin inactivation by adding 5 volumes of 10% FBS media, then washed with phosphate buffered saline (PBS), and incubated with the mouse antiserum from each group followed by staining with a PE-conjugated anti-mouse secondary IgG antibody (Jackson Immuno Research). Data acquisition and analysis were performed using LSRII (BD) and FlowJo (Tree Star) software.

### 2.7 ELISA

Sera from the animals were collected every two weeks and tested for MERS-S1 protein-specific IgG by conventional ELISA as previously described [12]. For long-lasting immunity, sera from the animals were collected at week 23 and week 55 after primary inoculation and tested for MERS-S1 protein-specific IgG by ELISA. Briefly, 96-well plates were coated with the serum-free media from A549 cells infected with 10 MOI of Ad5.MERS-S1 for 48 h overnight at 4°C in carbonating buffer (pH 9.5) and then blocked with PBS containing 0.05% Tween 20 (PBS-T) and 2% bovine serum albumin (BSA) for 1 hr. Mouse sera were diluted 1:200 in PBS-T with 1% BSA and incubated for two hours. After the plates were washed, anti-mouse IgG-horseradish peroxidase (HRP) (1:2000, SantaCruz) were added to each well and incubated for one hour. The plates were washed three times and developed with 3,3’5,5’-tetramethylbenzidine, and the reaction was stopped with 1M H_2_SO_4_ and absorbance at 450 nm was determined using an ELISA reader (PerkinElmer 2030). For the binding of the recombinant proteins and mouse sera against Ad5.MERS-S1, the 96-well plates were coated with 200 ng of the purified recombinant proteins per well overnight at 4°C in carbonate coating buffer (pH 9.5), followed by addition of diluted pre-immunized sera and sera from the mouse infected with AdΨ5 or Ad5.MERS-S1 [12], and carried out sequentially based on a protocol similar to that described above. For ELISA of SARS-CoV-2-S1 antibody, the 96-well plates were coated with 200 ng of the purified rSARS-CoV-2-S1 per well overnight at 4°C in carbonate coating buffer (pH 9.5), followed by addition of 1:100 diluted mouse sera, and carried out sequentially based on a protocol similar to that described above.

### 2.8 MERS-CoV neutralization assay

We tested the MERS-CoV neutralization activity of sera derived from mice immunized with rMERS-S1f only, rMERS-S1fRS09, rMERS-S1ffliC, MERS-S1f with MPLA, MNA-MERS-S1f, MNA-MERS-S1fRS09, MNA-MERS-S1ffliC. Ad5.MERS-S1, and PBS as previously described [12]. Mouse sera were obtained from the retro-orbital plexus every two weeks for six weeks and tested for their ability to neutralize MERS-CoV (EMC isolate). Briefly, virus (200 PFU) was premixed 1:1 with serial dilutions of sera from animal groups prior to inoculation onto Vero cells, and viral infection was monitored by the occurrence of a cytopathic effect at 72 h post-infection. Virus neutralization titers (VNTs) were determined as the highest serum dilutions that showed full protection against the cytopathic effect of MERS-CoV.

### 2.9 Statistical analysis

Statistical analyses were performed using GraphPad Prism (San Diego, CA). Data were analyzed by one-way ANOVA, followed by Tukey's post-hoc testing. Differences were considered significant if *p* < 0.05.

## 3 Results

### 3.1 Construction and characterization of recombinant proteins

**Fig. 1:**
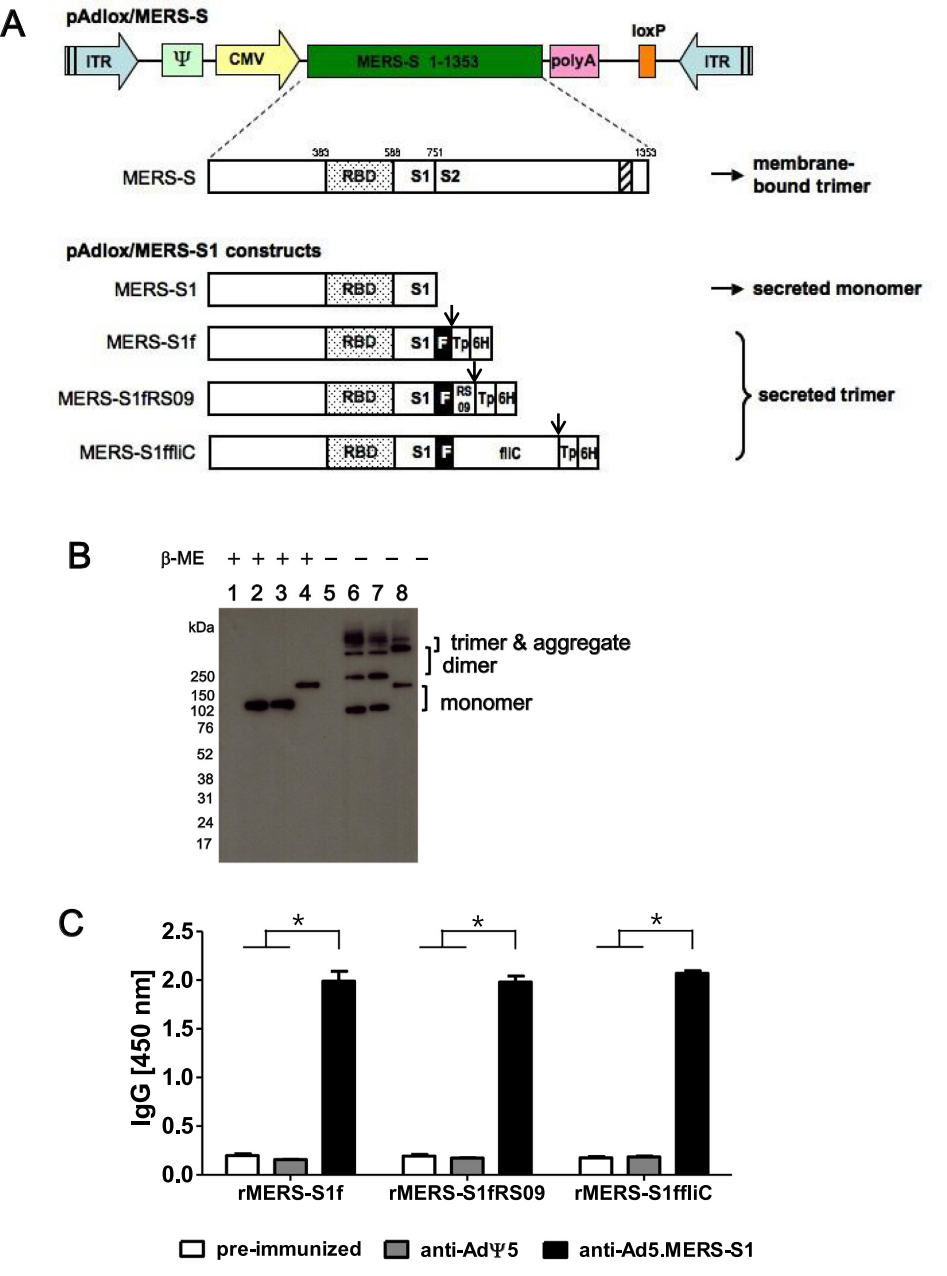
*Construction of recombinant MERS-S1 Foldon subunit vaccines***.** (A) Schematic diagram of rMERS-S1s. The positions of the RBD (small dots) and transmembrane domain (stripes) are indicated and S is divided into two subdomains, S1 and S2, at position 751. The vector was used to generate recombinant replication-deficient adenoviruses by homologous recombination with the adenoviral genomic DNA. Arrows showed the cleavage site by TEV protease. Abbreviations are as follows: ITR, inverted terminal repeat; RBD, receptor binding domain;F, T4 fibritin foldon trimerization domain; Tp, Tobacco Etch Virus (TEV) protease; fliC; Salmonella typhimurium flagellin C (B) Western blot of the supernatant of A549 cells infected (10 MOI) with mock (lane 1 and 5), Ad5.MERS-S1f (lane 2 and 6), Ad5.MERS-S1fRS09 (lane 3 and 7), or Ad5.MERS-S1ffliC (lane 4 and 8), respectively, with anti-6His monoclonal antibody. The supernatants were resolved on SDS-4~20% polyacrylamide gel after being boiled in 2% SDS sample buffer with or without β-ME. (C) Detection of purified rMERS-S1fs with mouse sera against Ad5.MERS-S1. The recombinant MERS-S1f proteins were purified using His60 Ni Superflow Resin under native conditions. After cleavage of the fusion protein by TEV protease, 6xHis tag and protease were removed from the cleavage reaction by affinity chromatography on a nickel chelating resin. Three rMERS-S1fs were coated in 96-well plate with 200ng/well ELISA and detected with mouse sera against Ad5.MERS-S1. Significance was determined by one-way ANOVA followed by Tukey's test. *p < 0.05.

To construct the recombinant trimeric MERS-S1 proteins, codon-optimized MERS-S1 was fused to the T4 fibritin foldon trimerization domain. Two additional variants were engineered by integrating the TLR4 agonist peptide (RS09) or Salmonella typhimurium flagellin C (fliC) fragment at the C-terminal of the foldon. Moreover, all three antigens were designed with a 6-histidine tag and a Tobacco Etch Virus (TEV) protease cleavage sequence at the carboxy terminus to allow for metal chelating affinity purification and to facilitate downstream large-scale, purification compatible with clinical manufacturing (Fig. 1A). A shuttle vector (pAd/MERS-S1f) was generated and a replication-defective adenovirus 5, designated as Ad5.MERS-S1f, was produced by loxP homologous recombination to use as a positive control. To determine whether MERS-S1f, MERS-S1fRS09, and MERS-S1ffliC would form trimeric structures, the serum-free supernatants from A549 cells infected with each adenovirus were characterized by SDS-PAGE and Western blot analysis. Three recombinant proteins designed with a 6-histidine tag at the carboxy-terminal ends were recognized by a monoclonal anti-6His antibody at the expected glycosylated monomer molecular weights of about 130 kDa, 135 kDa, and 200 kDa under the denaturing reduced (with β-ME) conditions. The higher molecular-weight bands (dimers or trimers) were evident under the denatured non-reduced (without β-ME) condition, and were 2 or 3 times that of the reduced proteins (monomer), as expected (Fig. 1B). [45,46] After being boiled in the sample buffer, the proteins still migrated to the position of the trimer, indicating the remarkable stability of these proteins. These results demonstrate that recombinant MERS-S1 formed a trimeric conformation in the presence of the Fd trimerization motif. In addition, to delete the six-histidine tag, the purified recombinant proteins were treated with AcTEV protease (Life Technology) followed by affinity chromatography on a nickel chelating resin to remove the six-histidine tags and poly-histidine tagged protease from the cleavage reaction. The cleaved native recombinant proteins were collected from the flow-through fraction and used as coating antigens followed by detection with mouse serum. Three purified recombinant proteins were detected using serum from mice immunized with Ad5.MERS-S1 (*p*<0.05, one-way ANOVA, followed by Tukey's post-hoc testing) prepared in our laboratory previously [12], while no specific antibody binding activity was detected in the serum of pre-immunized naïve mice or from control mice inoculated with AdΨ5 (Fig. 1C). ELISA data revealed that the three recombinant MERS-S1 proteins had strong reactivity with MERS-S1-specific antibodies.

### 3.2 Detection of membrane-bound MERS-S-specific antibodies

**Fig. 2:**
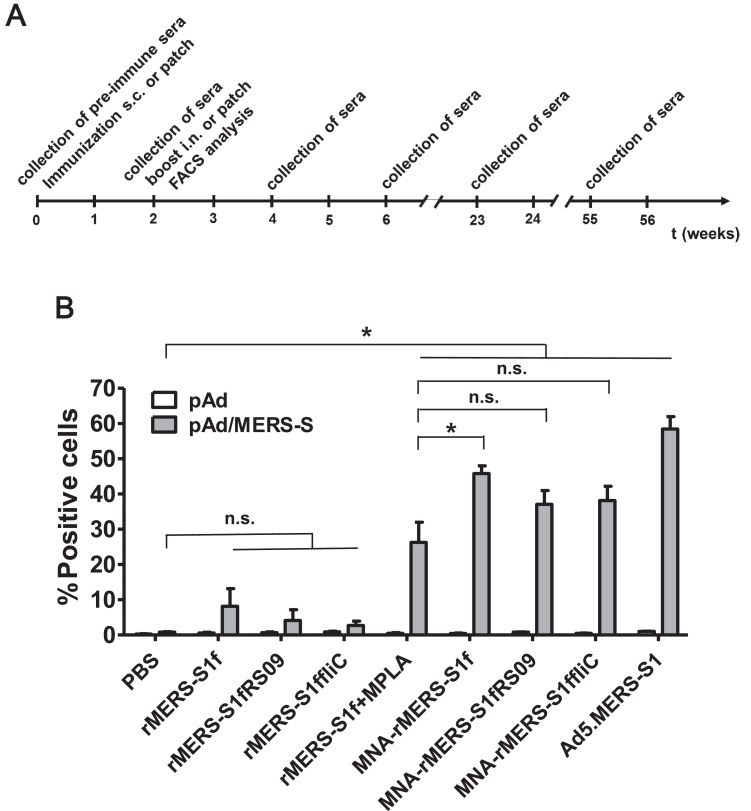
**MNA delivered subunit vaccines elicit antibodies recognizing cell surface bound MERS-S proteins.** (A) The experimental schedule representing the timeline for the immunizations. (B) Flow cytometry assay of 293 cells expressing MERS-S at the cell surface. HEK 293 cells were transfected with pAd/MERS-S (gray bar) or control pAd (white bar). At 36 h post-transfection, binding to MERS-S at the cell surface was analyzed by incubation with mice sera obtained at week 2 after immunization followed by staining with PE-conjugated anti-mouse IgG. St Significance was determined by one-way ANOVA followed by Tukey's test. *p < 0.05.

We next determined whether these recombinant subunit vaccines could elicit antigen-specific immune responses *in vivo*. BALB/c mice were inoculated subcutaneously with 25 µg of MERS-S1f, 25 µg of MERS-S1fRS09, 40 µg of MERS-S1ffliC, or 25 µg of MERS-S1f plus 20 µg of the MPLA adjuvant to deliver the same molar ratio of immunogen, or PBS as a negative control on day 0. The experimental schedule is shown in Fig. 2A. Two weeks after immunization, we obtained sera and examined binding to membrane-bound MERS-S by measuring the reactivity against 293 cells transfected with pAd/MERS-S or pAd using flow cytometry. As shown in Fig. 2B, in the presence of MPLA adjuvant, rMERS-S1f induced a strong membrane-bound MERS-S-specific IgG antibody response (p<0.05, one-way ANOVA, followed by Tukey's post-hoc testing), while no significant antibody response was detectable in the mouse sera of rMERS-S1f, rMERS-S1fRS09, and rMERS-S1ffliC, when compared with PBS-immunized mouse sera. On the other hand, in the absence of the adjuvant sequence, MNA intracutaneous delivery of MERS-S1f, MERS-S1fRS09, or MERS-S1ffliC, at the same molar ratio, elicited potent IgG antibody responses (p<0.05, one-way ANOVA, followed by Tukey's post-hoc testing) that were significantly stronger than those seen in s.c. immunized mice with MPLA adjuvant (p<0.05, one-way ANOVA, followed by Tukey's post-hoc testing), indicating the potential of MNAs as an effective recombinant coronavirus vaccine delivery platform. (Fig. 2B). Mice immunized with Ad5.MERS-S1 as a positive control developed significantly higher levels of IgG antibody against cell membrane-bound MERS-S as compared to negligible IgG levels in control mice inoculated with PBS. Antibodies were not detected bound to cells transfected with pAd without the immunogen insert (data not shown). These data indicate that MERS-S1f protein with MPLA adjuvant and MNA delivered rMERS-S1f, rMERS-S1fRS09, and rMERS-S1ffliC induced strong antibody response to membrane-bound MERS-S two weeks after a single immunization.

### 3.3 Induction of humoral immune response to MERS-S1

**Fig. 3:**
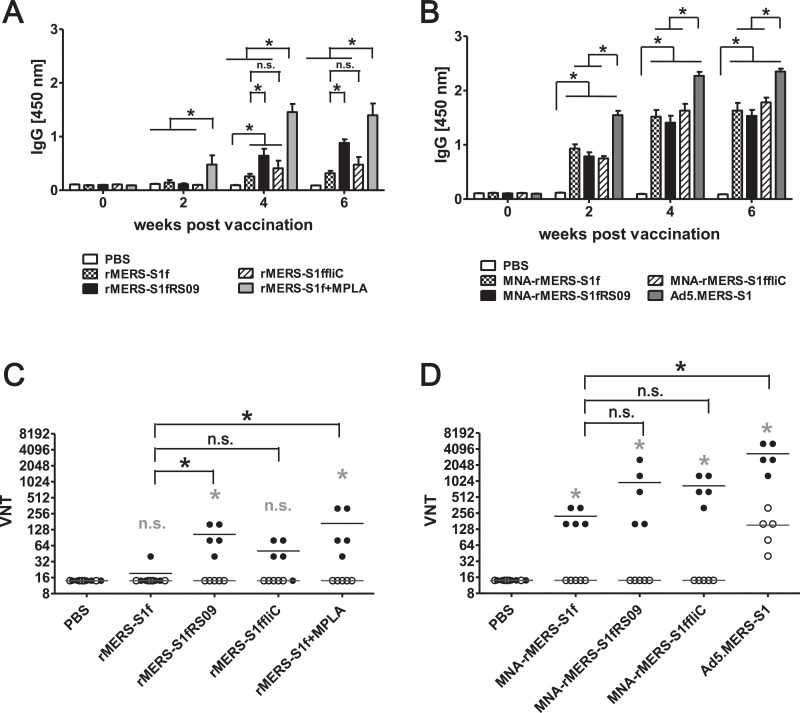
**Induction of humoral immune response in mice vaccinated with recombinant MERS-S1 vaccines.** BALB/c mice were immunized subcutaneously or intracutaneously with 20 µg of each rMERS-S1 subunit vaccines s.c., with or without 20 µg of MPLA, or by MNA delivery of the same subunit proteins, or with 10^11^vp of Ad5.MERS-S1 as a positive control. On day 14 mice were boosted using the same regimen as the prime immunization. On weeks 0, 2, 4, and 6 after treatment, immune sera from mice were collected, diluted (200x), and tested for the presence of MERS-S1-specific antibodies by ELISA (A and B) or by MERS-CoV-neutralization assay without dilution (C and D). MERS-CoV virus-neutralizing titers (VNTs) were measured every week after primary immunization using Vero cells by determining the highest dilution inhibiting MERS-CoV infection by 100%. Significance was determined by one-way ANOVA followed by Tukey's test. *p < 0.05.White and black circles in Fig. 3C and D represent week 4 and week 6, respectively.

To investigate the immunogenicity of trimeric MERS-S1f proteins, at two and four weeks after a boosting immunization, sera were obtained from all mice and screened for MERS-S1 specific antibodies by ELISA. As shown in Fig. 3A, following s.c. vaccination only Ad5.MERS-S1 elicited a MERS-S1-specific IgG antibody response (*p*<0.05, one-way ANOVA, followed by Tukey's post-hoc testing) at week 2, while no antibody response was detectable in the mouse sera of rMERS-S1f, rMERS-S1fRS09, and rMERS-S1ffliC vaccinated mice when compared with PBS-immunized mouse sera. However, 2 weeks after boosting, antigen-specific IgG was detected in the sera of all s.c. immunized mice, although the antibody titers were generally low. As an exception to this, s.c. delivered MERS-S1fRS09 induced a relatively strong MERS-S1-specific IgG antibody response (p<0.05, one-way ANOVA, followed by Tukey's post-hoc testing, at weeks 4 and 6). Importantly, mice immunized with each of the vaccine variants by MNA delivery demonstrated significantly higher levels of antigen-specific IgG (*p*< 0.05, one-way ANOVA, followed by Tukey's post-hoc testing) as compared to mice immunized with PBS, and there was with no significant differences between the MNA vaccine variants (Fig. 3B).

To further demonstrate the immunogenicity of MERS-CoV subunit vaccines, mouse sera were also tested for their ability to neutralize MERS-CoV (EMC isolate). As shown in Fig. 3C, there were no detectable MERS-CoV-neutralizing antibodies in the sera of mice immunized s.c. with MERS-S1f-, MERS-S1fRS09-, or MERS-S1ffliC at week 4. However, at week 6, sera of animals immunized with rMERS-S1fRS09, rMERS-S1ffliC, or rMERS-S1f + MPLA had significant and comparable levels of virus neutralizing activity, with geometric mean neutralizing titers of 104, 50.8, and 168, respectively. These titers were 5.4, 2.6, and 8.8 fold higher than that of sera from the mice immunized with MERS-S1f only (VNT mean, 19.2). Most importantly, as shown in Fig. 3D, at week 6 all groups of MNA immunized mice developed significant levels of neutralizing antibodies (*p*< 0.05, one-way ANOVA, followed by Tukey's post-hoc testing). Animals immunized with MNA-rMERS-S1fRS09, MNA-rMERS-S1ffliC, and Ad5.MERS-S1 had geometric mean neutralizing titers of 960, 832, and 3328, respectively. As expected, no neutralizing activity was detected in the sera of mice immunized with PBS. These results suggest that MNA delivery of these candidate subunit vaccines generates strong antibody-mediated neutralizing activity that approaches that induced by live adenovector immunization and exceeds that observed by s.c. delivery, even when a potent exogenous adjuvant is added to the s.c. vaccine. The promising immunogenicity of MNA MERS-CoV vaccines without chemical adjuvants makes them an ideal candidate for future clinical studies.

### 3.4 Longevity of the immune response in mice vaccinated with subunit vaccines

**Fig. 4:**
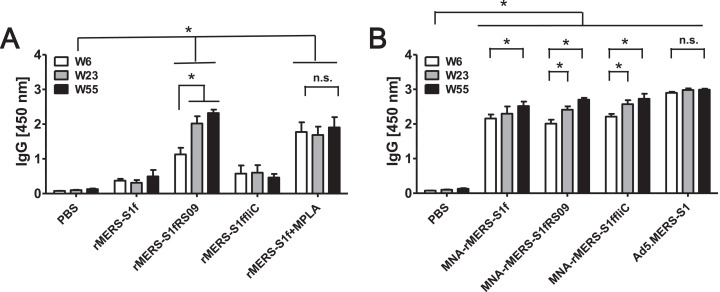
**Longevity of immune response in mice vaccinated with subunit vaccines.** BALB/c mice were immunized with subunit vaccines as described in the Fig. 3 legend. On week 23 and 55 after immunization sera were collected, diluted (200x) in PBS, and tested for the presence of MERS-S1-specific antibodies by ELISA. Significance was determined by one-way ANOVA followed by Tukey's test. *p < 0.05.

To evaluate the persistence of MERS-S1-specific immunity, mouse sera was collected at weeks 23 and 55 after immunization and evaluated for the presence of MERS-S1-specific IgG by ELISA. All animal groups immunized s.c. with recombinant subunit vaccine demonstrated the same or more levels of MERS-S1 IgG specific antibodies at the later time points as those observed at week 6. Surprisingly, mice immunized s.c. with rMERS-S1fRS09 had significantly higher levels of IgG (*p*< 0.05, one-way ANOVA, followed by Tukey's post-hoc testing) at week 23 compared with those observed at week 6 (Fig. 4A), and this was sustained through 55 weeks. Importantly, MNA delivered vaccines demonstrated generally increasing (statistically significant) levels of MERS-S1-specific antibody through the 23- and 55-week time points (Fig. 4B). No significant difference was observed in the mice immunized with PBS or Ad5.MERS-S1 at any time point evaluated. These results indicate that MNA-delivered subunit vaccines induce potent and long-lasting MERS-S1 specific antibody responses and MNA delivery is a viable approach for the clinical development of subunit vaccines against emerging infectious diseases such as COVID-19.

### 3.5 Immunogenicity of MNA delivered SARS-CoV-2-S1 subunit vaccines

**Fig. 5:**
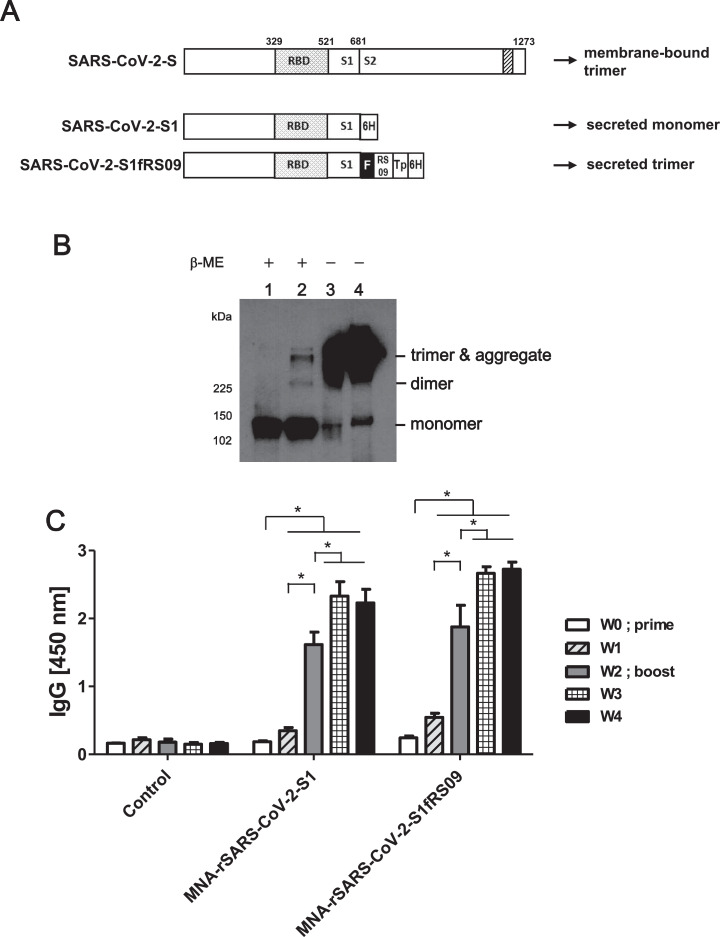
**Induction of humoral immune response in mice immunized with MNA delivered SARS-CoV-2-S1 vaccines.** (A) Schematic diagram of rSARS-CoV-2-S1s. The positions of the RBD (small dots) and transmembrane domain (stripes) are indicated and S is divided into two subdomains, S1 and S2, at position 681. The SARS-CoV-2-S1 or SARS-CoV-2-S1fRS09 gene was inserted into pmax Cloning expression vector. (B) Western blot of the purified rSARS-CoV-2-S1 (lane 1 and 3) or rSARS-CoV-2-S1fRS09 (lane 2 and 4) proteins with anti-6His monoclonal antibody. The purified proteins were resolved on 10% Tris/Glycine gel after being boiled in 2% SDS sample buffer with β-ME or in native sample buffer without β-ME. (C) C57BL/6 mice were immunized intradermally with MNAs containing 20 µg of rSARS-CoV-2-S1 or rSARS-CoV-2-S1fRS09. Immune sera from mice were collected every week, diluted (100x) in PBS, and tested for the presence of SARS-CoV-2-S1-specific antibodies by ELISA. Significance was determined by one-way ANOVA followed by Tukey's test. *p < 0.05.

Based on these MERS-S1 vaccine results and the urgency of the public health threat by COVID-19, we focused our efforts on the development of SARS-CoV-2-S1 and SARS-CoV-2-S1fRS09 subunit vaccines (shown in Fig. 5A). The size and trimerization product of the vaccine was confirmed by western blot (Fig. 5B). The aggregation in Fig. 5B could be attributed to the trimerization of the S1’s segments NTD, CTD1, and CDT2 domains or the ability of the histidine tag to oligomerize the tagged proteins [[47], [48], [49]]. We used MNAs to vaccinate mice with SARS-CoV-2-S1 and SARS-CoV-2-S1fRS09 subunit vaccines. Sera was collected prior to immunization (week 0) and at weeks 1 2, 3, and 4, and evaluated for the presence of SARS-CoV-2-S1 specific antibodies by ELISA as previously described. As shown in Fig. 5C, significantly high (*p*< 0.05, one-way ANOVA, followed by Tukey's post-hoc testing) SARS-CoV-2 IgG responses were detected as early as week 2 for all subunit vaccines as compared to control groups. The inclusion of the RS09 ligand in the trimer subunit resulted in a negligible effect on SARS-CoV-2 specific IgG titers. Further, in our preliminary study, the immunogenicity of gamma irradiation sterilized MNA vaccines was comparable to that of unirradiated MNA vaccines (Data not shown), thereby supporting the feasibility of gamma irradiation as a terminal sterilization approach for our clinical MNA-SARS-CoV-2 subunit vaccines.

### 3.6 Rapid development of clinically applicable MNA SARS-CoV-2 S1 subunit vaccines

Relying on our experience with MERS-CoV and SARS-CoV vaccines, and our experience with clinical production and human applications of MNAs, we developed standard operating procedures (SOPs) for the rapid development of clinic grade MNA SARS-CoV-2 subunit vaccines. Our production strategy and the related timeline is outlined in Table 1. First, we designed, optimized, and cloned the S1 subunits beginning when the sequence became publicly available. Subsequently, we produced S1 subunit proteins, purified them, and then fabricated dissolvable MNAs integrating the protein subunits for pre-clinical testing in the animal model. Next, we vaccinated mice with MNA-rSARS-CoV-2 vaccines and began analysis of antibody responses induced in immunized animals. These preclinical studies were initiated within 4 weeks of the SARS-CoV-2 spike sequence becoming publicly available. In parallel, to enable future regulatory approval and rapid clinical testing, we employed Good Manufacturing Processes (GMP) using a certified master cell bank (MCB) to scale S1 subunit protein production, purification, and validation, and incorporated the subunits into dissolvable MNAs or syringes using processes developed through our clinical production and quality control of MNAs (ClinicalTrials.gov Identifier: NCT02192021). This was followed by (1) gamma irradiation to assure sterilization of the vaccine loaded MNAs and syringes that will be used as control groups in our clinical trials, and (2) analysis of established release criteria, the latter of which is now ongoing and will be used for an application for IND approval that will enable a safety focused phase I clinical trial.

**Table 1:** Timeline for the development of SARS-CoV-2 subunit vaccines.

## 4 Discussion

Here, we present the rapid design and translational development of MNA SARS-CoV-2 vaccines capable of generating potent antigen-specific antibody responses by capitalizing on our substantial experience with Coronavirus vaccines and MNA delivery platforms.

First, we describe the design and production of trimeric recombinant subunit vaccines against MERS-CoV-S1 with and without integrated immunostimulatory TLR ligand sequences. We tested the immunogenicity of these vaccine variants delivered either by traditional subcutaneous needle injection or using MNAs to more specifically target vaccine components to the immune fertile skin microenvironment. We found that MNA delivery of MERS-CoV-S1 vaccines induced stronger humoral responses than traditional needle injections regardless of the inclusion of TLR ligand binding sequences (Fig. 3A,B). On the other hand, integration of the RS09 TLR4 binding sequence in the subunit trimer resulted in relatively stronger antibody responses than those without RS09 when vaccines were delivered by subcutaneous injection (Fig. 3A). Interestingly, the inclusion of the flagellin TLR5 binding sequence had no effect on the immunogenicity of the s.c. delivered vaccines (Fig. 3B). Importantly, significant neutralizing activity was observed in vaccinated animals at week 6. Consistent with previous IgG results, the presence of RS09 in the subunit trimer improved the neutralization function of antibodies from s.c. immunized mice, and this was the only immunogen that resulted in significant neutralization. Notably, MNA delivery was more effective in eliciting neutralizing antibodies for all vaccines. All MNA vaccines generated higher levels of neutralizing antibody, even beyond those induced by an s.c. delivered MPLA adjuvanted vaccine, and similar to those seen following the delivery of the rMERS-S1f construct via recombinant adenoviral vector. Though levels of neutralizing antibody were elevated in mice immunized with trimers including either RS09 or flagellin, these increases were not statistically significant when compared to the MNA vaccine alone (Fig. 3C,D). Thus, though the contributions of the integrated TLR ligands to the strong immune responses induced by MNA vaccines appear to be minimal, they provide promising benefit for s.c. vaccine administration, and their potential for contribution to immunogenicity in humans has yet to be determined. Importantly, we and others have shown effective protection against MERS-CoV challenge in hDPP4-expressing mice at comparable or lower neutralizing titers in previous passive immunotherapy studies with MERS-CoV-specific antibodies or neutralizing sera obtained from MERS-CoV immune humans or animals [[50], [51], [52], [53]]. Furthermore, we have previously demonstrated that antibodies directed against S1 domain of the spike protein, as detected in our study by ELISA, exert neutralizing activity against live coronaviruses [54].

Based on our results with MERS-CoV-S1 vaccines, we focused our efforts to develop MNA SARS-CoV-2 vaccines. Using the described approach, within three weeks of publication of the SARS-CoV-2- spike glycoprotein sequence, we produced both rSARS-CoV-S1 and rSARS-CoV-S1fRS09 immunogens and fabricated dissolvable MNAs incorporating these vaccines in quantities sufficient for pre-clinical testing. MNA delivery of either rSARS-CoV-2-S1 or rSARS-CoV-2-S1fRS09 induced substantial and statistically significant increases in antigen-specific antibodies responses as soon as week 2 compared to pre-immunization and week 1 responses. The inclusion of RS09 did not have a significant effect on antibody titer at the 2, 3, or 4-week time points. Furthermore, the immunogenicity of MNA vaccines was maintained after gamma irradiation sterilization, suggesting a viable method to assure sterility of our MNA vaccines for clinical applications. The significant antibody titers we observed at the early time points before boosting strongly supports the feasibility of our MNA-SARS-CoV-2 vaccines, particularly in the context of similar results obtained with the analogous MERS-CoV-S1 constructs under the same conditions.

Neutralization assays are important to ensure antibody function; however, at this early timepoint, we do not have access to validated assays for neutralizing antibodies against SARS-CoV-2. Further, at the time of submission of this manuscript, we are just over four weeks post-immunization and based on our MERS-CoV studies, the reliable detection of neutralizing SARS-CoV-2 antibodies may require at least six weeks post immunization. We speculate that this delay in the neutralizing response is likely a result of the time needed for affinity maturation of the SARS-CoV-2 neutralizing IgG antibodies. However, we believe that MNA-SARS-CoV-2 vaccines will very likely induce neutralizing immunity as suggested by the vigorous early antibody responses, and commonalities between both the vaccines and the viruses. Accordingly, even though it is still early to predict whether humans immunized with these vaccine candidates will have similar responses and be protected from SARS-CoV-2 or MERS-CoV infections, our studies demonstrate that development, production, and initial animal testing of clinically translatable MNA vaccine candidates against SARS-CoV-2 and other emerging infections can be rapidly accomplished. Studies directly assessing important correlates of human efficacy would provide additional support for efficacy in humans. In this regard it will be important to determine whether antibodies from MNA-SARS-CoV-2 immunized animals will neutralize virus infectivity. Further, the demonstration of protection from infection in animal challenge models, such as the hACE2 transgenic mouse model, would be informative. We plan to do these studies when these models are developed and validated. Finally, we note that the immunogenicity differences between MNA coronavirus vaccines and coronavirus vaccines delivered by traditional needle injection that we observe will need to be evaluated in clinical trials to establish the clinical advantages of MNA delivery.

Microneedle array mediated immunization has several mechanistic differences from traditional intramuscular or subcutaneous needle injections, which could explain the variations in the magnitude and kinetics of the ensuing responses [[55], [56], [57], [58], [59]]. The skin is immunologically reactive and contains a high density of antigen presenting and immune-accessory cells with innate immune function including keratinocytes, mast cells, and innate lymphocytes [25,[60], [61], [62]]. Redundant skin immunoregulatory circuits can respond to a wide variety of damage or infection related signals to rapidly orchestrate an innate immune response [[62], [63], [64]]. Further, MNAs deliver vaccine components to a defined 3D space within the skin microenvironment which results in very high vaccine concentrations with relatively low dose antigen delivery. MNA delivery of antigens to targeted skin microenvironments results in prolonged exposure of skin resident antigen presenting cells and other innate immune cells to the skin delivered components [65]. Moreover, transient mechanical stress from microneedle insertion can induce a natural local innate immune response which can serve as a physical adjuvant to enhance antigen-specific adaptive immunity [57]. MNA delivery of lower doses to a localized 3D space improves safety by reducing systemic exposure [27]. Further, MNA delivery has the potential to accelerate the process of vaccine production and to significantly reduce cost by considerably reducing the required vaccine doses [58]. The stability studies with our MNA SARS-CoV-2 vaccines are currently in progress, however; there is evidence in the literature that vaccine components including proteins are typically stabilized by integration into the MNA polymer matrix, and retain their conformational structures, as evidenced by maintenance of antibody binding function [29,66] or retained immunogenicity of recombinant adenovirus vaccines for at least a month at 25 °C [67]. Thus, MNA vaccines offer the potential to eliminate the substantial costs associated with the “cold chain” necessary for current vaccines [34,68]. Finally, the MNAs described here are designed to be applied without the need for an applicator or any specialized equipment, supporting the potential for self-administration. As such, these features provide several important advantages that support the future development of MNA vaccines for global protection from rapidly emerging infectious diseases.

Taken together, our studies demonstrate the speed at which vaccines against emerging infections can be designed and produced using the recent advances in recombinant DNA technology. Combining emerging biotechnology methods with bioengineering advances in vaccine delivery strategies, it may now be possible to rapidly produce clinically-translatable vaccines against novel pathogens for human testing and subsequent global distribution in time to significantly impact the spread of disease.
